# Tunable-Porosity Membranes From Discrete Nanoparticles

**DOI:** 10.1038/srep17353

**Published:** 2015-12-02

**Authors:** Patrizia Marchetti, Martin Mechelhoff, Andrew G. Livingston

**Affiliations:** 1 Department of Chemical Engineering, Imperial College London South Kensington Campus, SW7 2AZ, London UK; 2 Lanxess Deutschland GmbH Chemiepark Krefeld-Uerdingen, 47829 Krefeld-Uerdingen, Germany

## Abstract

Thin film composite membranes were prepared through a facile single-step wire-wound rod coating procedure in which internally crosslinked poly(styrene-co-butadiene) polymer nanoparticles self-assembled to form a thin film on a hydrophilic ultrafiltration support. This nanoparticle film provided a defect-free separation layer 130–150 nm thick, which was highly permeable and able to withstand aggressive pH conditions beyond the range of available commercial membranes. The nanoparticles were found to coalesce to form a rubbery film when heated above their glass transition temperature (T_*g*_). The retention properties of the novel membrane were strongly affected by charge repulsion, due to the negative charge of the hydroxyl functionalized nanoparticles. Porosity was tuned by annealing the membranes at different temperatures, below and above the nanoparticle T_*g*_. This enabled fabrication of membranes with varying performance. Nanofiltration properties were achieved with a molecular weight cut-off below 500 g mol^−1^ and a low fouling tendency. Interestingly, after annealing above T_*g*_, memory of the interstitial spaces between the nanoparticles persisted. This memory led to significant water permeance, in marked contrast to the almost impermeable films cast from a solution of the same polymer.

The macromolecular architecture of the top separating layer of nanofiltration membranes is crucial to their function in molecular separations. Nanofiltration membranes are formed via interfacial polymerization, coating, or the phase inversion processes and the resulting separating layer nanostructure is strongly dependent on the fabrication methods and polymer chemistry^1^,^2^.

In integrally skinned asymmetric membranes prepared via the phase inversion process, spherically shaped polymer nodules form at the top surface, providing a nanoporous separating layer^3^. The nodule diameters are usually in the range 50–100 nm and are strongly influenced by the physico-chemical properties of the polymer and the composition of the dope solution. It is difficult to predict and characterize the properties of the polymer nodules in these top layers and the porosity gradient along the asymmetric membrane thickness. As the resulting nodular membrane is in a non-equilibrium state, thermal annealing can cause significant changes in flux and separation factor, through densification of the separating layer. During this process, the nodular structure observed in the non-annealed membrane is replaced with a continuous non-porous dense layer interspersed with nodules, and the membrane separating layer undergoes a gradual loss of nanoporosity^3^. In thin film composite (TFC) membranes, a chemically different thin film which acts as the separating layer is fabricated on top of a more open support^4^,^5^. This allows independent control of the separating layer to achieve a desired flux and rejection, and the porous support for mechanical strength and minimum resistance to permeate flow^1^. The separating layer is usually prepared by interfacial polymerization, creating a thin polyamide film, while the support-layer typically consists of an ultrafiltration (UF) membrane, most commonly polysulfone, formed by phase inversion. The porosity of the resulting separation layer is a function of a large number of chemical and process variables, including the monomers used to form the polyamide, the reaction conditions for polymerization, and the properties of the ultrafiltration support. The inherent complexity of both phase inversion and interfacial polymerization fabrication processes means that is it is not presently possible to correlate the structural properties of these membranes with their functional performance.

More reproducible fabrication methods for polymer membranes with well-defined surface chemistry and pore sizes at a few nanometers would be highly attractive for NF membrane manufacturing^6^. Seeking to achieve this, an alternative to phase inversion and interfacial polymerization based on colloidal assembly of polymeric nanoparticles, via a so-called “bottom-up” approach, has been advanced^7^. This approach exploits the organization of nanomaterials, which act as pre-structured building blocks, to create a separating layer with tunable properties. Crosslinked organic nanoparticles have defined structural properties and can be processed in an aqueous solution (a latex, or nanofluid) without the need for an organic solvent, hence providing an environmentally friendly production process. This approach offers potential advantages including: (i) the control of membrane structure at the nanoscale by creating highly ordered arrays of monodisperse particles^8^,^9^; (ii) the control of surface properties via appropriate *in-situ* particle functionalization during synthesis, and; (iii) the possibility of using crosslinked polymer networks with high durability towards oxidizing agents or in solutions of extreme pH, conditions which usually lead to a rapid breakdown of conventional polyamide-based membrane materials. Within the past decade interest in using colloidal polymer particles to create mambranes has been growing. Membranes with UF and microfiltration (MF) properties were fabricated by depositing polymer nanoparticles made of polystyrene and various acrylates with diameters from 80 to 980 nm onto the surface of a microporous substrate. The resulting porous array was fabricated by repeated filtrations, required to fill cracks in the first layer by a naturally greater flow through the defective areas, and stabilized by heating at temperatures up to 150 °C^9^. Employing particles of smaller size to establish a mean pore size and fill the gaps between bigger particles was also attempted^10^. However, mesoporous multilayers of polystyrene nanoparticles with average particle diameters between 20 and 80 nm fabricated via dead-end filtration techniques suffered from substantial cracking upon drying, leading to large defects in the membrane top layer^11^. Recently, ultrathin and mechanically strong porous separation membranes of cross-linked polystyrene nanoparticles with uniform thickness were prepared^6^, using a sacrificial layer made of metal hydroxide nanostrands on a polycarbonate microporous support. Nanoparticles with diameter from 15 to 50 nm and modified with amino groups were filtered onto the support membrane and cross-linked afterwards. Filtration had to be repeated, because the small nanoparticles could not form a complete array and further filtration was required to ensure the large pores were filled selectively. The formation of nanoparticle arrays was also attempted for applications in organic solvents^12^. UF membranes were prepared by assembling 120 nm and 300 nm large polymer particles with methacrylate moieties onto the surface of cross-linked polyimide UF support membranes. After spin-coating from a methanol solution, the nanoparticles were cross-linked by a photo-initiated free radical polymerization using ultraviolet light. Considering this previous work, it is apparent that while researchers have been successful in preparing particle-based membranes which show micro- or ultrafiltration properties, nanofiltration membranes remain challenging. This is because the size of the particles required results in films that suffer from cracks and defects upon drying; and so, elaborate multi-step coating methods for repair are necessary. These multi-step methods make the approach difficult for larger scale scale up to industrial production. Nevertheless, there remains an interesting parallel between integrally skinned asymmetric nanofiltration membranes and nanoparticle nanofiltration membranes. During phase inversion, it is not yet possible to control the polymer nodule size which dictates the nanostructure of the separating layer of the membranes which are formed. If it were possible to form the separating layer from nodule-size nanoparticles, one might deliberately create a similar structure in a more controlled manner. This is the path we have followed in this work. A nanofiltration membrane was prepared through a facile single-step coating procedure using internally crosslinked poly(styrene-co-butadiene) polymer nanoparticles (equivalent to those used in^13^) on top of a hydrophilic UF support. The separating layer formed of nanoparticles created a highly permeable and selective separation layer with NF properties, useful for the treatment of industrial process and waste waters. The nanoparticles possess high chemical and mechanical stability under extreme conditions, such as concentrated acid and base solutions and a wide range of organic solvents^13^, and can therefore be applied in aggressive conditions where currently available commercial membranes would fail quickly. The performance of these membranes was evaluated using solutes of different sizes and charges, and the mechanisms of size exclusion and surface charge and their effects on the membrane performance are fully discussed. The structural properties of the membrane were characterized by microscopy and zeta potential measurements. Interestingly, upon heating above the nanoparticle glass transition temperature (T_*g*_), the particles coalesced, forming a rubber-like apparently dense film. The permeance and rejection performance of the resulting semi dense film which retains “memory” of the original nanoparticle structure was compared to that of a rubbery film formed from linear polymers, which usually has insignificant water permeance^14^,^15^.

## Results and Discussion

### Membrane fabrication

**Figure 1:**
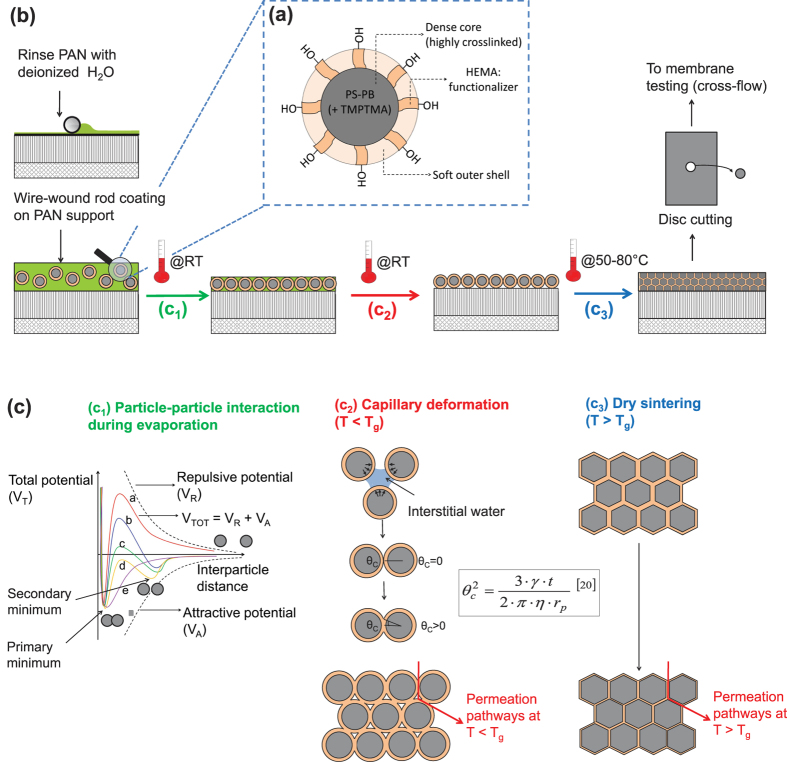
(**a**) Schematic representation of the hydroxyl functionalized core-shell poly(styrene-co-butadiene) nanoparticles. PS = polystyrene; PB = polybutadiene; TMPTMA = trimethylolpropane trimethacrylate, as a crosslinker; HEMA = 2-hydroxyethyl methacrylate, as a hydroxyl functionalizer. (**b**) Schematic representation of the preparation process for TFN membrane via wire-wound rod coating on polyacrylonitrile (PAN) support. Surface loading of nanoparticles used for the rod coating was in the range of 66 to 198 mg_*NP*_ m^−2^_membrane_. (**c**) Physical phenomena occurring during nanoparticle deposition and film formation are schematically displayed in: (c_1_) Particle-particle interaction and approach during evaporation of the bulk water; (c_2_) capillary deformation by particle-particle coalescence during evaporation of the interstitial water (temperature (T)< glass transition temperature (T_*g*_)); (c_3_) dry sintering at T > T_*g*_. The equation for the half-angle of contact between two particles, 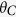, is from Brown^20^. 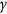 is the liquid surface tension, *t* the time, 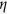 the polymer viscosity, and r_*p*_ the particle radius.

Thin film nanocomposite (TFN) membranes were prepared via a wire-wound rod coating procedure on a polyacrylonitrile (PAN) support, as shown schematically in Fig. 1. The latex dispersion consisted of crosslinked, *in-situ* hydroxyl functionalized core-shell poly(styrene-co-butadiene) nanoparticles, suspended in an aqueous solution (see Fig. 1(a)). The modification with hydroxyl groups rendered the nanoparticles more hydrophilic than an unmodified styrene-butadiene copolymer. The nanoparticles used in this study had a diameter of 48.0 ± 0.5 nm (see Supplementary Information, Figure S1) and a glass transition temperature, T_*g*_, of 63.2 °C (see Supplementary Information, Figure S2). The dispersion was puddled in front of the wire-wound rod at one end of the ultrafiltration support, and drawn across the surface with the rod moving over it. Provided the support material was sufficiently wetted by the aqueous solution, a latex film formed on the surface. Upon drying of the film (by water evaporation) the nanoparticles arranged themselves in a layer of ordered arrays with interstices between the particles, interconnected and so providing permeation pathways. A schematic representation of the preparation process for TFN membrane via wire-wound rod coating on polyacrylonitrile (PAN) support is shown in Fig. 1(b).

The PAN support was chosen for its mechanical, chemical and thermal durability^16^. Furthermore, due to its relatively high surface energy, PAN has good wettability for aqueous solutions, which ensures a uniform spreading of water on the surface during coating. No post-treatment with preserving agents is required for the PAN support to be mechanically and chemically stable. The PAN support was prepared on a polypropylene non-woven by a phase inversion procedure, as described in the Supplementary Information, Figure S3. Wettability and water permeance of the PAN support are shown in the Supplementary Information, Figure S4.

In colloidal suspensions, the interaction potential between two nanoparticles is dominated by the contributions of an attractive Van der Waals potential (V_*A*_), decaying strongly with increasing distance, and a repulsive electrostatic potential (V_*R*_), originating from the overlap of the particle double layers^17^. The total potential energy of interaction between two nanoparticles is given by the sum of the attractive and the repulsive contributions: V_*T*_ = V_*A *_+ V_*R*_, as described by the Derjaguin-Landau-Verwey-Overbeek (DLVO) theory (see Fig. 1(c_1_))^17^. During deposition of the polymer colloid, a principally regular structure was formed by the assembly of the monodisperse nanoparticles used as the “building blocks”^8^. The evaporation rate is responsible for bringing the particles together and has an effect on the film quality: fast evaporation causes an increase in the interparticle capillary pressure, which may lead to cracking^5^, and a fast increase in the solution viscosity^18^, which causes slower particle mobility and less time for particle rearrangement. As less defects and grain boundaries appear at relatively slower evaporation rates^5^,^18^,^19^, a slow water evaporation at a room temperature (RT) of 23–25 °C and a relative humidity of 32–36% was allowed for all membranes in this work.

**Figure 2:**
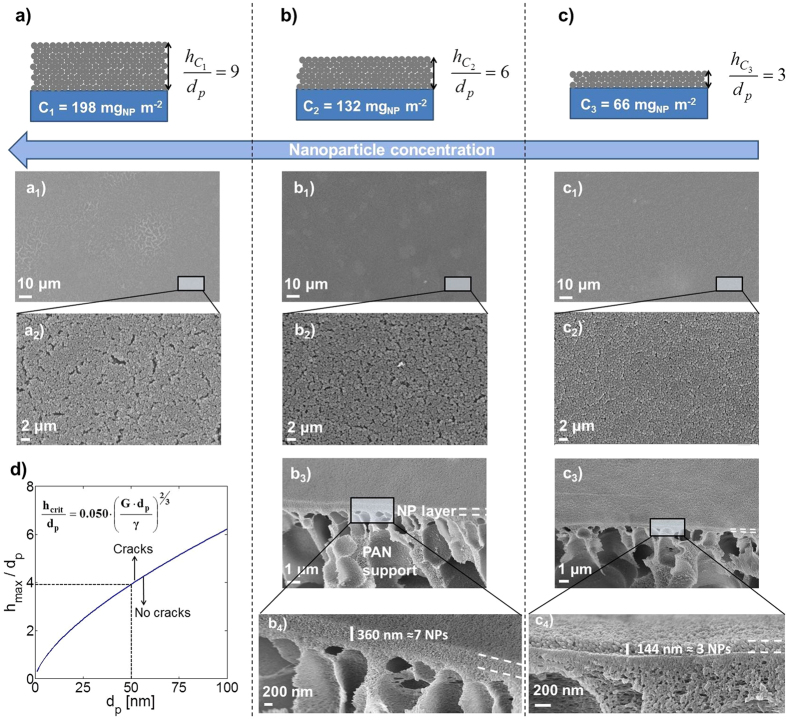
SEM surface images of TFN membranes at different magnifications on porous PAN supports. The films were prepared via wire-wound rod coating with different nanoparticle surface loadings and then dried at room temperature. (a_1_–a_2_) 198 mg_*NP*_ 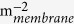; (b_1_–b_4_) 132 mg_*NP*_ 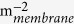; (c_1_–c_4_) 66 mg_*NP*_ 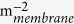. (**d**) Profile of the critical film thickness (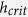 for each particle size, 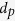), from ref. 21.

Whether or not a dense film is formed by the interpenetration of the outer shells of the discrete nanoparticles depends on the temperature of the deposition process relative to the nanoparticle glass transition temperature (T_*g*_) (see Fig. 1(c_2_,c3)). The T_*g*_ is dependent on the viscoelastic resistance of the polymer to pass from a hard and relatively brittle state into a soft, rubber-like state. For the nanoparticles in this study, after coating and drying at room temperature, an array of discrete particles formed with interstices clearly visible in SEM imaging. This is shown in Fig. 2(a–c) for TFN membranes prepared using three different nanoparticle surface loadings.

Figure 2(a–c) shows that, at temperatures below T_*g*_, the particles did not lose their discrete structure and remained spherical. No nanoparticle intrusion in the porous PAN support was observed. In addition, the nanoparticle layer adhered tightly to the PAN support, since it withstood the surface drag of cross flow membrane testing. This was probably due to the presence of van der Waals forces and hydrogen bonds between the hydroxyl surface groups of the particles and the PAN surface. The membrane prepared with the highest nanoparticle surface loading of 198 mg_*NP*_ m^−2^_membrane_ showed significant cracks formation (see Fig. 2(a_1_,a_2_)), while the membrane prepared with the lowest nanoparticle surface loading of 66 mg_*NP*_ m^−2^_membrane_ showed the most uniform surface (see Fig. 2(c_1_,c_2_)). The nanoparticle surface loading had an effect on the film thickness, with a film thickness of 360 nm given by a nanoparticle surface loading of 132 mg_*NP*_ m^−2^_membrane_ (see Fig. 2(b_3_,b_4_)) and a film thickness of 144 nm given by a nanoparticle surface loading of 66 mg_*NP*_ m^−2^_membrane_ (see Fig. 2(c_3_,c_4_)).

Together with a very low DLVO interaction potential, another reason why soft nanoparticles form a stable film (i.e. a film in which the nanoparticles do not redissolve in the original liquid medium, once deposited and dried) is the partial interpenetration of their outer shells. This partial coalescence occurs as a consequence of capillary deformation driven by interstitial capillary pressure (see Fig. 1(c_2_))^20^. According to Brown^20^, during water evaporation, particles become ordered to acquire the most favorable arrangement. Interdiffusion of polymers across the particle-particle boundaries takes place, driven by the capillary pressure generated across the water/air menisci. The capillary pressure and the extent of particle deformation correlate with the half-angle of contact between two particles, 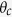, to the liquid surface tension, 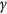, the time t, the polymer viscosity 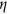, and the particle radius r_*p*_^20^. The capillary pressure, however, has a critical value, above which particles are stressed, exceeding their limit of deformation. This leads to the development of cracks in the film. At a constant and fixed evaporation rate, the most significant parameter that can affect cracking is the film thickness, which in turn correlates with the nanoparticle surface loading in the original latex solution. Russel *et al.*^21^ reported the effect of nanoparticle surface loading on cracking, providing a correlation for the critical film thickness (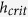) per particle size (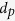) above which cracking in the film can be observed, as shown in Fig. 2(d). For a particle diameter of ∼50 nm, h_*crit*_/d_*p*_ ∼4. This is in agreement with our observation of uniform film formation for membrane (c) in Fig. 2 (theoretical h_*crit*_/d*_p_* = 3), and more widespread cracking at higher nanoparticle surface loadings, such as for membrane (a) in Fig. 2 (theoretical h_*crit*_/d_*p*_ = 9).

The experimental film thickness measured by scanning electron microscopy (SEM), was compared with the theoretical film thickness, calculated assuming that the film is a colloidal crystal with a hexagonal, or honeycomb, packing (which corresponds to the crystalline structure with highest packing density for equal spheres). A good agreement between the theoretical thickness of the colloidal crystal and the actual thickness of the colloidal glass was found, as shown in Table 1. Finally, the film porosity of the packed bed of spheres was calculated using both Leva’s and Ergun’s equations. The former predicted a slightly higher porosity than the latter (0.095/0.098 vs. 0.080/0.084), but interestingly both calculations indicated a porosity independent of the membrane thickness.

After partial particle interdiffusion at T < T_*g*_ due to capillary stress, the membrane was heated at T > T_*g*_ and subjected to dry sintering (driven by the reduction of the polymer/air interfacial energy upon heating)^8^. This step is schematically presented in Fig. 1(c_3_). During coalescence by dry sintering, the particles deformed, thus filling all available space, and the boundaries between the particles flattened to form a more dense structure. During dry sintering, the center of mass of the nanoparticle does not change upon polymer relaxation. A theoretically possible complete coalescence is however prevented by the highly crosslinked nature of the nanoparticle core, which remains intact upon interpenetration of the less crosslinked outer shells (see Supplementary Information, Appendix A.1).

**Figure 3:**
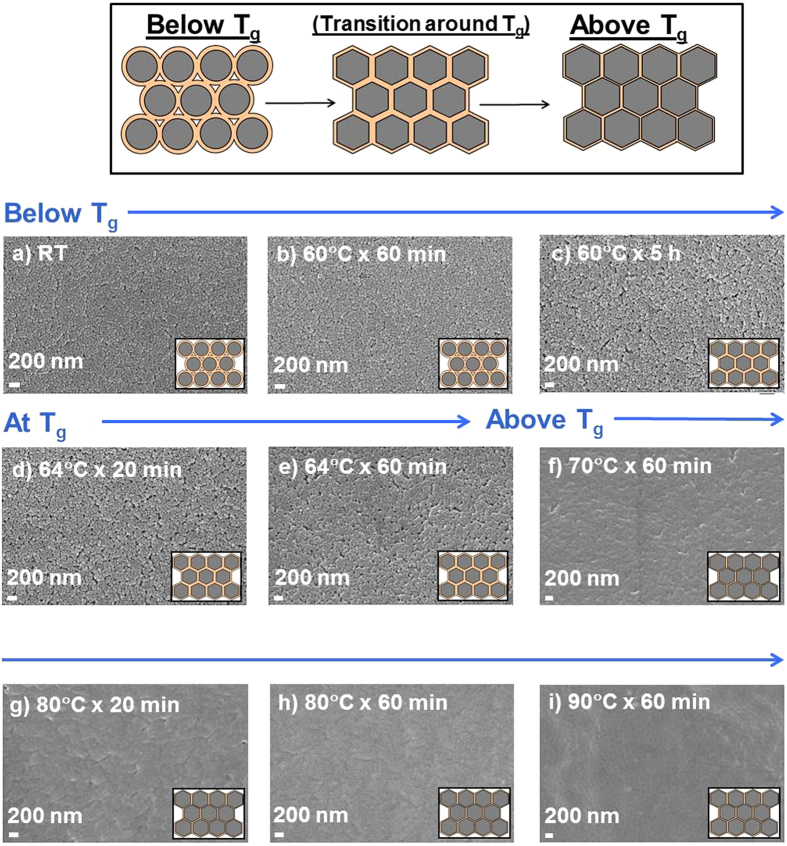
SEM surface images of TFN membranes under different heating conditions on porous PAN support. The films were prepared via wire-wound rod coating with a nanoparticle surface loading of 66 mg_*NP*_ m^−2^_membrane_. The membranes were dried at room temperature (RT) for at least 24 h and then heated in the oven at different temperature for varying times. (**a**) no heat treatment (RT); (**b**) 60 °C for 60 min; (**c**) 60 °C for 5 h; (**d**) 64 °C for 20 min; (**e**) 64 °C for 60 min; (**f**) 70 °C for 60 min; (**g**) 80 °C for 20 min; (**h**) 80 °C for 60 min; (**i**) 90 °C for 60 min.

The TFN membranes were heated at different temperatures, below, at, and above the nanoparticle T_*g*_, for varying times. The corresponding SEM images of the membrane surfaces are shown in Fig. 3.

Figure 3 shows that the nanoparticles maintain their discrete structure below the nanoparticle T_*g*_, while, above the nanoparticle T_*g*_, they coalesce with the neighboring particles. At 60 °C, heating time has no effect on particle annealing (see Fig. 3(a–c)), while, at 64 °C, heating for 60 or 20 minutes gives a slightly smoother surface (see Fig. 3(d,e)). After 60 minutes, the particles start to interpenetrate, visible from a tightening of membrane pores. Above the nanoparticle T_*g*_, the gaps within the original nanoparticles are no longer visible in the SEM pictures (see Fig. 3(f–i)), implying that the outer shells became interpenetrated. The membrane surface is generally smooth, as shown by atomic force microscopy (AFM), reported in Table 2. The root-mean-square roughness (R_*ms*_) is between 4.97 and 5.12 nm and the average roughness (R_*a*_) is between 3.95 and 4.20 nm for membranes dried at room temperature and 60 °C, hence below the nanoparticle T_*g*_. However, both R_*ms*_ and R_*a*_ values were reduced to about 3 and 2.3 nm, respectively, for membranes dried at 70 °C and 80 °C, showing a layer of strongly interpenetrated nanoparticles. This implies that there is a noticeable effect of heating temperature on the surface roughness due to particle coalescence, with the dense membranes being the smoothest.

### Membrane performance

**Figure 4:**
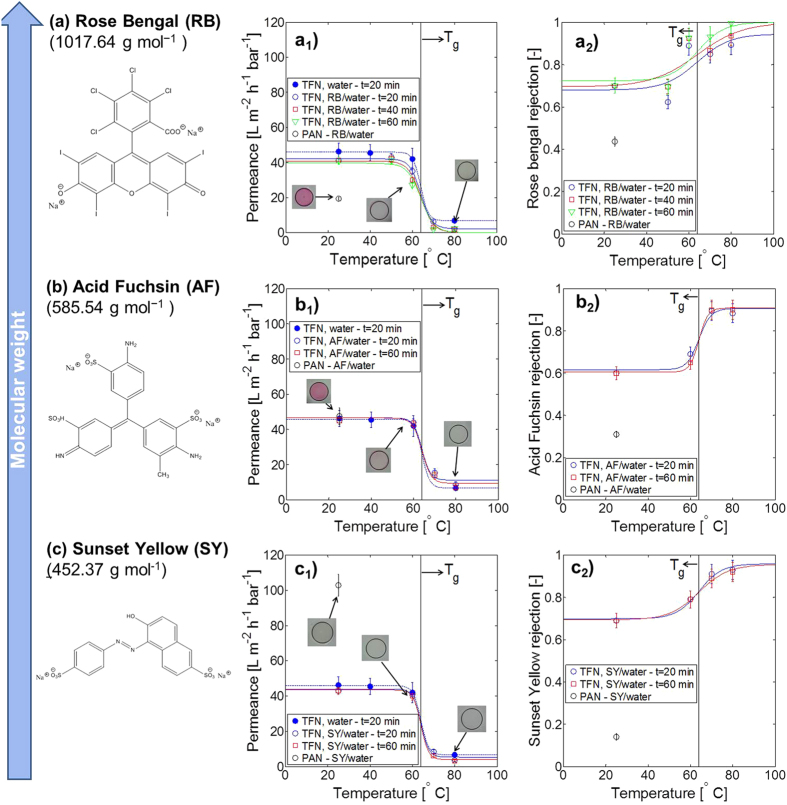
Performance of TFN membranes on porous PAN supports with different dyes in water (20 mg L^−1^). The films were prepared via wire-wound rod coating with a nanoparticle surface loading of 66 mg_*NM*_ m^−2^_membrane_. The membranes were dried at room temperature for at least 24 h and then heated in the oven at different temperatures for varying times. (**a**) Rose Bengal/water; (**b**) Acid Fuchsin/water; (**c**) Sunset Yellow/ water. The membranes were tested in a cross-flow system, with constant cross-flow rate of 100 L h^−1^, pressure of 10 bar and room temperature. The permeance and rejection profiles were fitted with a hyperbolic tangent model (the hyperbolic tangent model parameters are reported in Supplementary Information, Table S1).

The performance of the TFN membranes was initially tested with aqueous solutions of various dyes (Rose Bengal, Acid Fuchsin and Sunset Yellow, in decreasing order of molecular weight). Dyes were used to represent organic contaminants of typical industrial waste water, e.g. in the textile industry. The membrane performance is shown in Fig. 4 in terms of permeance and solute rejection.

Fig. 4 shows permeance (on the left hand, see Fig. 4(a_1_,b_1_ and c_1_)) and rejection (on the right hand, see Fig. 4(a_2_,b_2_ and c_2_)) of nanoparticle-based TFN membranes using the following markers: Rose Bengal in Fig. 4(a), Acid Fuchsin in Fig. 4(b) and Sunset Yellow in Fig. 4(c). For all the solutions, permeance decreased from ca. 40 L m^−2^ h^−1^ bar^−1^ to ca. 10 L m^−2^ h^−1^ bar^−1^ and rejection increased to values between 0.9 to 1 with increasing heating temperature. Neither parameter showed a significant dependence on the heating time, varied from 20 to 60 minutes. The corresponding uncoated PAN support showed rejection values of ca. 0.15 to 0.45, depending on the solute type. Hence, coating of the PAN support with the aforementioned nanoparticle layer increased membrane rejection remarkably, and produced membranes with tight ultrafiltration properties. Further, heating of the particle layer above the nanoparticle T_*g*_ yielded a membrane with a nanofiltration molecular weight cut-off below 500 g mol^−1^. We assert this is due to dry sintering upon heating above the nanoparticle T_*g*_, which causes particle-particle fusion and the reduction of permeation pathway size. A statistical analysis on the effect of temperature and heating time on both permeance and rejection is reported in Supplementary Information, Table S2.

The permeance of the uncoated PAN support in the dye solutions is reported in Fig. 4(a_1_, b_1_ and c_1_) (black circles), to understand the effect of the nanoparticle layer on the overall performance. Interestingly, the relationship between the permeance of the uncoated PAN support and that of the TFN membrane is different for the different dyes, and is a function of the dye molecular size. In the presence of Rose Bengal, the permeance of the PAN support at room temperature is lower than that of the coated membrane at the same temperature (see Fig. 4(a_1_)). In the presence of Acid Fuchsin, the two permeance values are comparable (see Fig. 4(b_1_)), while in the presence of Sunset Yellow (see Fig. 4(c_1_)) the permeance of the PAN support at room temperature is significantly higher than that of the coated membrane. For all three dyes, the rejection was higher for the TFN membranes than for the uncoated PAN support. In absolute terms, the rejection was higher for Rose Bengal (see Fig. 4(a_2_)), followed by Acid Fuchsin (see Fig. 4(b_2_)) and finally Sunset Yellow (see Fig. 4(c_2_)). The relative behaviour of the uncoated PAN support vs. the TFN membrane was analyzed accounting for both permeance and rejection at the same time, and explained in terms of the different fouling tendencies of the two membranes. Rose Bengal was found to be a very strong foulant for the uncoated PAN support, as also visually demonstrated by the intense pink color of the membrane after use (see Fig. 4(a_1_)). As a consequence of strong fouling, flux was lower and rejection higher than for the other dyes. This could be related to both a higher molecular affinity between Rose Bengal and the PAN surface, and the bigger molecular size of Rose Bengal. Acid Fuchsin has an intermediate behaviour, with a lower fouling tendency than Rose Bengal (the slightly pale pink colour of the membrane after use is shown in Fig. 4(b_1_)); this results in higher flux for the uncoated PAN and lower dye rejection with respect to Rose Bengal. The smallest dye, Sunset Yellow, was found to be a very weak foulant for the PAN support, as the PAN had a large permeance (of more than 100 L m^−2^ h^−1^ bar^−1^), a very low dye rejection (of 0.15), and an almost white colour after use (see Fig. 4(c_1_)). The fouling tendency of the TFN membranes was much lower than that of the uncoated PAN support, as is clear from the photographs in the corresponding figures on the left hand of Fig. 4. This could be explained by the occurrence of stronger repulsion interactions between the hydroxyl groups on the membrane surface, resulting from the original nanoparticle functionalization, and the negative charge of the dye anions in solution. The performance of the TFN membranes was tested also after soaking in 1 M HCl/water and 1 M NaOH/water for 24 hours. The nanoparticle layer was resistant to these conditions, and maintained good flux and retention performance (see Supplementary Information, Figure S6).

**Figure 5:**
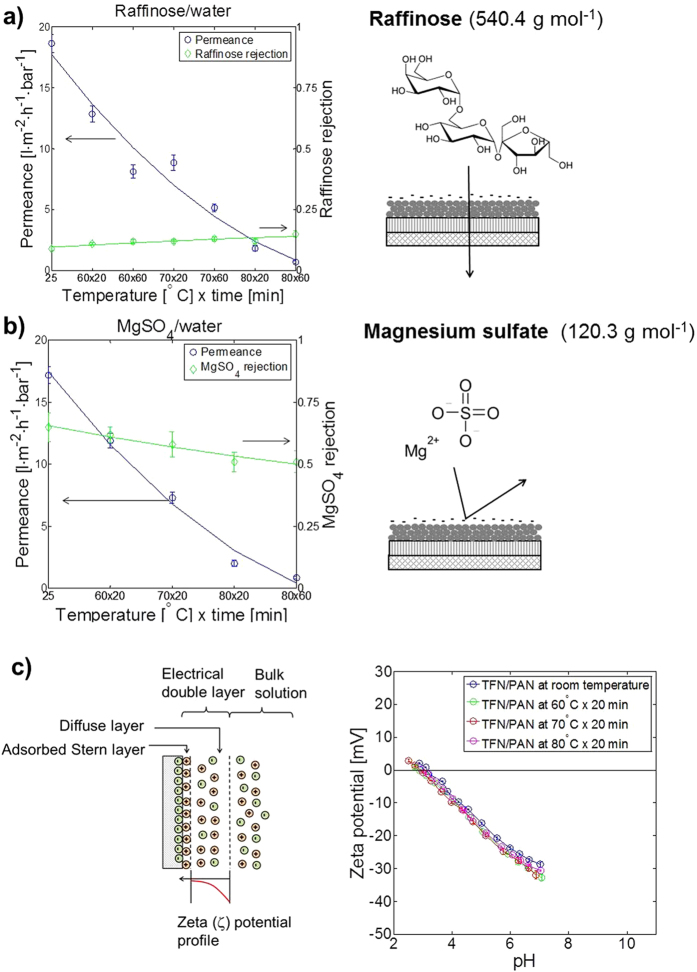
Performance of TFN membranes on porous PAN support with neutral and charged solutes of low molecular weight. The films were prepared via wire-wound rod coating with a nanoparticle surface loading of 66 mg_*NP*_ m^−2^_membrane_. The membranes were dried at room temperature for at least 24 h and then heated in the oven at different temperatures for varying times. The membranes were tested in a cross-flow system, with constant cross-flow rate of 100 L h^−1^, pressure of 10 bar and at room temperature. (**a**) 100 mg L^−1^ raffinose/water; (**b**) 100 mg L^−1^ MgSO_4_/water. (**c**) Schematic representation of electrical double layer formed at the membrane surface and zeta potential of membrane at acidic pH, measured by the streaming potential technique.

Comparing the figures on the right hand side of Fig. 4, it is interesting to observe that the rejection does not show a significant dependence on the molecular weight of the different dyes, while it shows a dependence on the permeance (see Supplementary Information, Figure S7). This implies that steric retention is not the main mechanism governing the transport through these membrane. Since the membrane is characterized by hydroxyl groups due to the OH-containing functionalization during synthesis of the nanoparticles, and all three dyes form negatively charged anions when dissolved in water, it can be concluded that the retention mechanism of these novel membranes is governed by the negative surface charge of both solute and membrane surface. To further test the membrane performance and validate the hypothesis of significant charge contribution to the overall rejection, two other model solutes were used: a small neutral solute, raffinose, with a molecular size similar to the dyes used before, and a charged salt of low molecular weight, magnesium sulfate (MgSO_4_). The former was used to identify the contribution of steric retention without charge effects, the latter to identify the effect of charge without significant steric interaction. The performance of the TFN membranes with these two solutes in water is shown in Fig. 5(a,b).

In the presence of both raffinose and MgSO_4_, permeance decreased significantly with increasing heating temperature (see Fig. 5(a,b), respectively). The neutral raffinose showed very low rejection, slightly increasing with a rising heating temperature (see Fig. 5(a)). MgSO_4_, on the other hand, showed higher and almost constant rejection values (see Fig. 5(b)). The heating temperature showed a significant effect on both flux and raffinose rejection, while MgSO_4_ rejection was constant over the studied range (see Supplementary Information, Table S2). The TFN membrane dried at room temperature (25 °C on the x-axis in Fig. 5(a,b)) has a lower rejection of 0.09 for raffinose, with a molecular weight of 540.4 g mol^−1^, than 0.65 for the charged MgSO_4_ salt, with a molecular weight of 120.3 g mol^−1^.

It is generelly accepted that the electrical double layer (EDL), which forms when a charged solid surface is in contact with an aqueous solution, is responsible for the charge rejection mechanism (see Fig. 5(c))^17^. The Zeta potential, 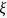, defined as the electrostatic potential at the boundary dividing the adsorbed layer and the diffuse layer, was deduced from streaming potential measurements of TFN membranes under four different conditions: non-annealed (dried at 25 °C), and annealed at 60, 70 and 80 °C for 20 minutes. The profile of the membrane Zeta-potential as a function of pH between pH 2.5 and 7 is shown in Fig. 5(c). The isoelectric point of the membrane was found to be at pH 3. Similar Zeta potential profiles were obtained for the four different membranes. This means that there is no significant effect of the annealing temperature, and hence the membrane porosity, on the surface charge, supporting the observation of a negligible effect of membrane porosity on magnesium sulfate rejection (see Fig. 5(b)). A negative potential of −35 mV at neutral pH implies that the membrane is strongly negatively charged under those conditions. It is therefore evident that the charged solute Acid Fuchsin showed a higher rejection than the neutral solute raffinose (comparing Fig. 4(d) and 5(a)), due to a charge repulsion mechanism at the membrane surface. Although both solutes have a similar molecular weight, size exlcusion did not play a role here.

**Figure 6:**
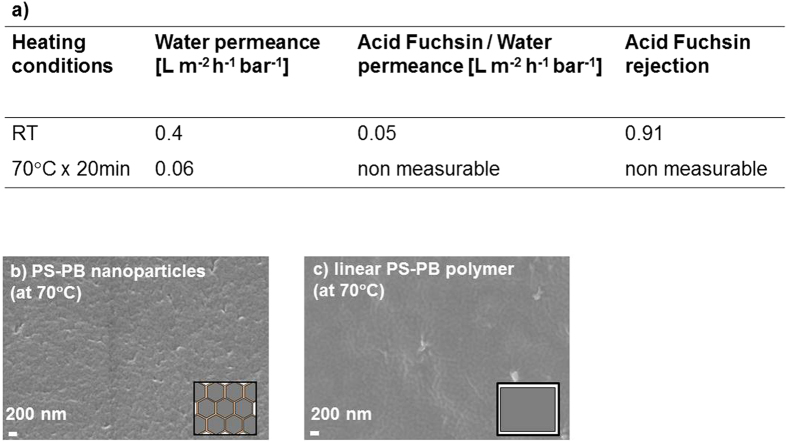
(**a**) Performance of dense poly-(styrene-co-butadiene) membranes on porous PAN support with Acid Fuchsin dye in water. The films were prepared via dip coating with a polymer concentration in water of 2.2 wt%. The membranes were dried at room temperature for at least 24 h and annealed under different conditions. (**b**) SEM surface image of TFN membrane on porous PAN support. The film was prepared via wire-wound rod coating with a nanoparticle surface loading of 66 mg_*NP*_ m^−2^ and dried at 70 °C for 60 min. (**c**) SEM surface image of dense poly-(styrene-co-butadiene) membranes on porous PAN support. The film was prepared via dip coating with a polymer concentration in water of 2.2 wt% and dried at 70 °C for 20 min.

Observing the unusually high water permeance of this novel rubber-like membrane, we speculated that the film formed upon annealing of the discrete nanoparticles at a temperature above T_*g*_ retains permeation pathways between the originally discrete nanoparticles, and in particular the dense crosslinked cores of these nanoparticles. In contrast, rubber-like films usually are almost impermeable to water. To test our speculation, a membrane was prepared via dip-coating of a solution of poly-(styrene-co-butadiene), the same polymer as is used to form the nanoparticles, dissolved in toluene, on top of PAN support at room temperature. Two different annealing conditions were used afterwards: no annealing (room temperature, RT) and annealing at 70 °C for 20 minutes, as reported in Fig. 6(a). The membrane thickness obtained using a solution of 2.2wt% polymer in toluene was 220 nm. Rejection and permeance of these membranes at 10 bar are reported in Fig. 6(a).

The pure water permeance of the non-annealed dense poly-(styrene-co-butadiene) membrane is 0.4 L m^−2^ h^−1^ bar^−1^ and hence about two orders of magnitude lower than the nanoparticle-based TFN membrane (ca. 40 L m^−2^ h^−1^ bar^−1^). The permeance further decreased to 0.05 L m^−2^ h^−1^ bar^−1^ in the presence of Acid Fuchsin, while still showing a significant rejection of 0.91. Annealing of the dense poly-(styrene-co-butadiene) membrane at 70 °C led to an even lower water permeance of 0.06 L m^−2^ h^−1^ bar^−1^, and no permeance at all in the presence of Acid Fuchsin. The Acid Fuchsin/water permeance of the annealed nanoparticle-based membrane was around 6 L m^−2^ h^−1^ bar^−1^, almost 100 times larger than that of the dense poly-(styrene-co-butadiene) membrane. This confirms that there is a significant difference between the two membranes, although they are chemically similar and have a reasonably similar thickness. Specifically, the nanoparticle-based membrane heated above T_*g*_ retains memory of the permeation pathways resulting from the interstitial regions between the dense cores of the original nanoparticles (see Fig. 6(b)), while the membrane from the linear polymer shows a dense and almost impermeable structure (see Fig. 6(c)). Thus one can conclude that the “bottom-up” approach permits the organization of nanomaterials with a desired chemical nature (in this study poly-(styrene-co-butadiene)) to form a membrane with desired properties, which may not be possible with the conventional coating methods from a polymer dope solution.

## Conclusions

In this work, TFN membranes based on the assembly of highly crosslinked poly(styrene-co-butadiene) nanoparticles were successfully fabricated by a single-step wire-wound rod coating on top of hydrophilic PAN UF support. No post-treatment with preserving agents was required for the composite membrane to be mechanically and chemically stable. During membrane fabrication, the nanoparticle surface loading was optimized, in order to avoid cracking during the drying step and obtain a thin, defect-free membrane separation layer of 144 nm thickness formed by discrete nanoparticles. The retention properties of the novel membrane were found to be strongly affected by charge repulsion, due to the highly negatively charged nature of the original nanoparticles. The high density of hydroxyl surface functionalization on the nanoparticles resulted also in low fouling tendency. Interestingly, upon heating above the nanoparticle glass transition temperature (T_*g*_), the particles coalesced, forming a rubber-like apparently dense film. The effect of drying time and temperature on the membrane performance was investigated and it was observed that the membrane porosity can be tuned by annealing the membranes at different temperatures. In this way it was possible to achieve nanofiltration properties with a molecular weight cut-off below 500 g mol^−1^. The nanoparticles comprise a highly crosslinked particle core and a more flexible and less dense outer shell, and we assert that the dense film formed as a consequence of deformation and interpenetration of the less dense outer shells. The result is a semi dense film which retains “memory” of the original nanoparticle structures, and specifically the highly crosslinked nanoparticle cores. The presence of permeation pathways within the nanoparticle-based membrane annealed above the film formation temperature was deduced from the observation of significant water permeance, compared to the almost impermeable nature of a dense membrane cast from a conventional rubber solution without spherical particles. The permeation pathways were found to be proportional to, and therefore tunable with, the annealing temperature. This provides the flexibility to fabricate different membranes with the same chemical composition and varying performance, as a function of the annealing conditions.

## Experimental Section

### Materials

Nanoparticles were supplied from Lanxess Deutschland GmbH, Cologne (Germany), as latex suspension in water, with a concentration of around 30%wt. They have a dense core (around 48 ± 0.5 nm in diameter) with extended polymer chains protruding from the surface. The end groups of the chains have -OH functionalities. Polyacrylonitrile (PAN) average Mw 150 000, sodium metabisulphite, different dyes (Rose Bengal, Acid Fuchsin and Sunset Yellow), raffinose and magnesium sulfate (MgSO_4_) were purchased from Sigma Aldrich, UK, and used as received. Non-woven polypropylene fabric was purchased from Novatexx, Germany, (product code 2471). Poly(styrene-co-butadiene), with 45%wt styrene content, was purchased from Sigma Adrich, UK. Dimethylformamide (DMF) and toluene was purchased from VWR, UK.

### Nanoparticle synthesis and characterization

The particles were synthesized by emulsion polymerization of butadiene, styrene and 2-hydroxyethyl methacrylate in water, according to the method described in^13^. For the experiments presented here, particles with 10.5 wt% butadiene, 80.5 wt% styrene and 7.5 wt% 2-hydroxyethyl methacrylate (HEMA) were used. 4 wt% trimethylolpropane trimethacrylate (TMPTMA) was used as a crosslinker, to achieve a very high degree of crosslinking. This typically produces particles with a gel content of >95% and a glass transition temperature of ca. 60 °C, hence being rather rigid at room temperature. The addition of HEMA led to a hydrophilic functionalization of the otherwise hydrophobic particles. During synthesis it was possible to tightly control the particle diameter to about 50 nm. Particle size and diffusivity of the actual particle batch used in this work were characterized by dynamic light scattering (DLS), using a Malvern Zetasizer Nanoseries ZEN1600. The glass transition temperature was measured by differential scanning calorimetry (DSC-7, Perkin-Elmer) using two heating ramps from −100 °C to +150 °C at a heating rate of 20 K min^−1^. Between the two measurements, a fast cool-down with 320 K min^−1^ was applied. The glass transition temperature was determined from a graphical interpretation of the DSC curve, with the value being set at half the variation of the specific heat coefficient between the beginning and the end of the glass transition.

### Preparation of polyacrylonitrile (PAN) support membrane

Support membranes were prepared from PAN via phase inversion. PAN powder was dissolved as received at room temperature in DMF (13 wt.% polymer/87 wt.% DMF) by means of mechanical stirring until an homogeneous solution was obtained and left overnight to allow the removal of air bubbles before its use. Membranes were cast on non-woven polypropylene fabric using the prepared dope solution on a continuous casting machine with the adjustable knife set at 250¼m and a speed of 0.035 m s^−1^. Immediately after casting, the membrane was immersed in a water bath (22 °C) where phase inversion occurred. After 10 min, membranes were transferred twice to fresh water baths and were finally stored in a 5 wt.% sodium metabisulphite solution. The procedure used to prepare PAN supports is schematically represented in the Supplementary Information, Figure S3.

### Preparation of Thin Film Nanocomposite (TFN) membrane

The TFN composite membranes were prepared using a K Control Coater and Paint applicator (RK Printcoat Instruments, UK). The wet PAN support was rinsed with water and taped on the coater to be kept flat. The nanoparticle solution was deposited using a meter bar (or wire-wound rod), manufactured by winding precision drawn stainless steel wire on to a stainless steel rod and therefore resulting in a pattern of identically shaped grooves. These grooves precisely control the wet film thickness. The meter bar used in this study has a wire diameter of 0.31 mm and delivers a wet film thickness of 24 *μ*m. The bar speed was fixed at 0.065 m s^−1^.

### Preparation of dense poly(styrene-co-butadiene) membrane

The membrane was cast from a solution containing poly-(styrene-co-butadiene) via dip-coating on top of PAN support at room temperature. The casting solution was prepared dissolving 2 g of poly(styrene-co-butadiene) in 100 mL of toluene. After casting, the toluene was allowed to evaporate at room temperature. Two different annealing conditions were used: no annealing (room temperature) and annealing at 70 °C for 20 minutes.

### Membrane characterization

TFN/PAN membranes were characterized by scanning electron microscopy (SEM) with a high resolution field emission gun scanning electron microscope (Carl Zeiss Ltd.) operating at 5 kV. Samples were coated with gold with a sputtered current of 20 mA for 0.5 min under an argon atmosphere to achieve the necessary conductivity. For the analysis of cross sections, samples were freeze-fractured in liquid nitrogen and coated.

Surface roughness was characterized by atomic force microscopy (Innova, Bruker). Samples were attached onto a magnetic sample disk using double sided adhesive tape and the scans were performed in an air medium. The images were scanned in tapping mode using phosphorous doped silicon tips (MPP-11100-W) with tip radius of less than 10 nm. Scanning was performed at a speed of 1 Hz, and a scan size of 1 *μ*m was used for standard images. A sampling resolution of 512 points per line was selected. Gwyddion 2.37 data visualization and analysis software was used to process the AFM images. Surface roughness is presented as average roughness (R_*a*_) and root-mean-square roughness (R_*ms*_).

The zeta potential of the membrane was obtained via streaming potential measurement on a EKA III (A. Paar) instrument. Ag/AgCL electrodes were used to measure the potential. The pressure drop in the cell was measured with a piezoresistive pressure transducer. The samples were inserted in the system wet and rinsed with water before the measurement. Conductivity and pH were also measured for each experiment. A titration unit was used to vary the pH in the measurement cell, from 2 to 7.

Contact angle measurements were performed on uncoated PAN supports with an Easy Drop Instrument (Kruess) at room temperature. The membranes were dried before the measurement and a drop of 15 *μ*L deposited on the surface. The contact angle was measured using a circle fitting method by the drop shape analysis software. Three measurements were done on each of three different discs.

### Nanofiltration Performance

**Figure 7:**
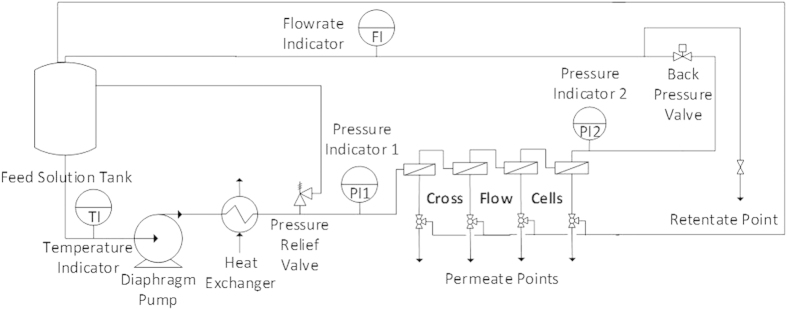
Schematic representation of the nanofiltration rig used to test the TFN membranes (reprinted with permission from Shi *et al.*^22^).

Membrane performance was determined by measuring the pure water flux and the rejection of aqueous solutions of three different dyes (Rose Bengal, Acid Fuchsin and Sunset Yellow), raffinose and MgSO_4_. Solvent flux and solute rejection were measured in a cross-flow filtration system at 5–10 bar for each nanoparticle-based TFN membrane. The rig used to test the membranes is shown in Fig. 7.

At least two membranes were tested each time to ensure reproducibility. The membrane discs had an area of 14 cm^2^. The pressure was set using a back pressure regulator located downstream of a pressure gauge. The temperature was kept at 30 °C using a heat exchanger. Due to the design of crossflow cells, the flow routine in the cells is spiral, from side to centre. However, the flow pattern is not well controlled as in pipes flow, therefore the velocity at the membrane surface is difficult to calculate. Considering the geometry of the cell in this study, the linear velocity for a feed flow rate of 100 L h^−1^ would result in 0.03 m s^−1^. During operation, permeate samples were collected from individual sampling ports and feed samples were taken from the feed tank. Rejection and flux (J_*V*_) were calculated according to Equations 1 and 2.


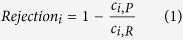



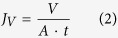



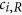
 and 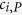 the concentrations at the retentate side and permeate side of the membrane, respectively, 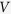 the volume collected in the permeate, *A* the membrane filtration area and 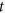 the permeation time. The concentrations of the feed solutions were 20 mg L^−1^ for dyes and 100 mg L^−1^ for raffinose and MgSO_4_. Concentration of dyes in retentate and permeate samples was measured by UV-vis spectrometry. UV spectra were recorded on a UV-1800 Shimazdu spectrophotometer in the range of 300–600 nm with a sampling interval of 0.5 nm. The absorption values at wavelengths of 545, 540 and 475 nm were used to calculate the rejection values of Rose Bengal, Acid Fuchsin and Sunset Yellow, respectively. An Agilent HPLC coupled to an evaporative light scattering detector (ELSD, Varian-385) was used to analyze raffinose concentration. The ELSD evaporator was set at 40 °C and the nebulizer at 55 °C. Nitrogen gas was supplied to the detector at a flow rate of 1.5 SLM (standard L m^−1^). A reverse phase column (C18-300, 250 mm 4.6 mm, ACE Hichrom) was used and the mobile phases were methanol and deionized water buffered with 0.1M ammonium acetate. The HPLC pump flow rate was set at 1 ml min^−1^ and the column temperature was set at 30 °C. Concentrations of MgSO_4_ were measured by conductivity meter (Hanna Instruments).

## Additional Information

**How to cite this article**: Marchetti, P. *et al.* Tunable-Porosity Membranes From Discrete Nanoparticles. *Sci. Rep.* **5**, 17353; doi: 10.1038/srep17353 (2015).

## Supplementary Material

Supplementary Information

## Figures and Tables

**Table 1:** Comparison of the SEM thickness of TFN membranes on porous PAN supports with the theoretical film thickness, if the films had a regular hexagonal (honeycomb) packing, and porosity values calculated using Leva and Ergun equations, respectively.

NP surface loading	SEM thickness	Calculated thickness^a^	Experimental water flux [L m^−2^ h^−1^ bar^−1^]	Porosity according to the Ergun equation^b^	Porosity according to the Leva equation^c^
66 mg_*NP*_ m^−2^_membrane_	144 nm (2.8 NPs)	125 nm (2.5 NPs)	44	0.095	0.084
132 mg_*NP*_ m^−2^_membrane_	360 nm (7.2 NPs)	250 nm (5 NPs)	19	0.098	0.080

The films were prepared via wire-wound rod coating on porous PAN supports with different nanoparticle surface loadings and then dried at room temperature.

^a^Assuming a hexagonal (honeycomb) packing arrangement.

^b^Ergun equation: 
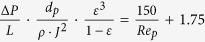
, 
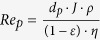

^c^Leva equation: 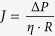, 
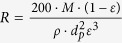
.

**Table 2:** Atomic force microscopy (AFM) roughness of selected membranes under different heating conditions on porous PAN support.

	RT	60 °C × 60m	64 °C × 20 m	70°C × 6 m	80° × 60 m
R_*sm*_ [nm]	4.97	5.12	5. 11	2.96	2.99
R_*a*_ [nm]	3.95	4.20	4.12	2.30	2.31

The films were prepared via wire-wound rod coating with a nanoparticle surface loading of 66 mg_*NP*_ m^−2^_membrane_. The membranes were dried at room temperature (RT) for at least 24 h and then heated in the oven at different temperature for varying times. Please refer to Supplementary Information, Figure S5 for AFM images of these membranes.
