# Supplementary material for: Tunable-Porosity Membranes From Discrete Nanoparticles
Source: Sci Rep. 2015 Dec 2;5:17353. doi: 10.1038/srep17353 (PMC4667292; doi:10.1038/srep17353)
Supplement: Supplementary Information [file srep17353-s1.pdf]

Supplementary Information:

Tunable-Porosity Membranes From Discrete Nanoparticles

by

Patrizia Marchetti[1], Martin Mechelhoff[2] and Andrew G. Livingston[1,*]

[1] Department of Chemical Engineering, Imperial College London
South Kensington Campus, SW7 2AZ, London (UK)

[2] Lanxess Deutschland GmbH
Chemiepark Krefeld-Uerdingen, 47829 Krefeld-Uerdingen, Germany

* to whom correspondence should be addressed. Tel +44-2075941210;

E-mail: a.livingston@imperial.ac.uk

A Supplementary Information

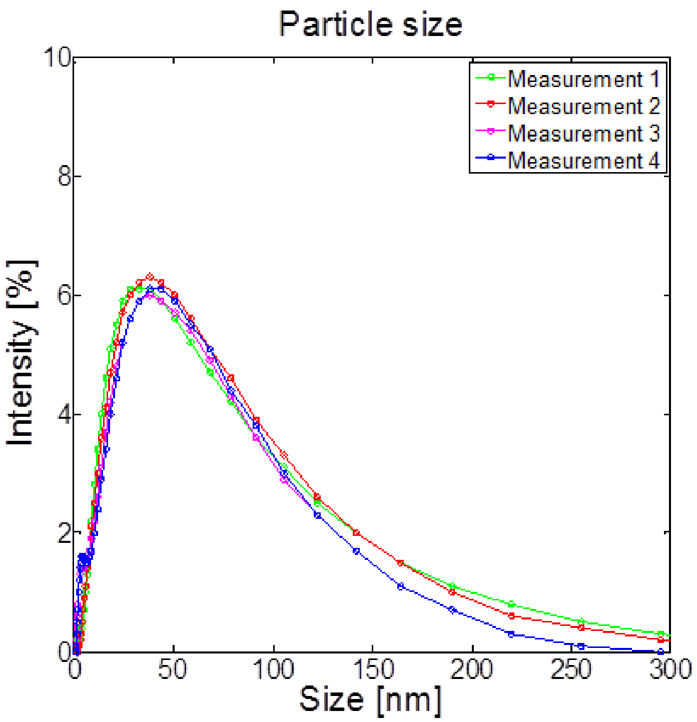

Measurement	Particle concentration [v%]	Peak mean [nm]	Polydispersity Index [-]
1	1.50	48.33	0.456
2	1.00	48.51	0.419
3	0.75	46.62	0.571
4	0.01	48.79	0.582

Figure S1: Particle size by dynamic light scattering (DLS) at various latex concentrations.

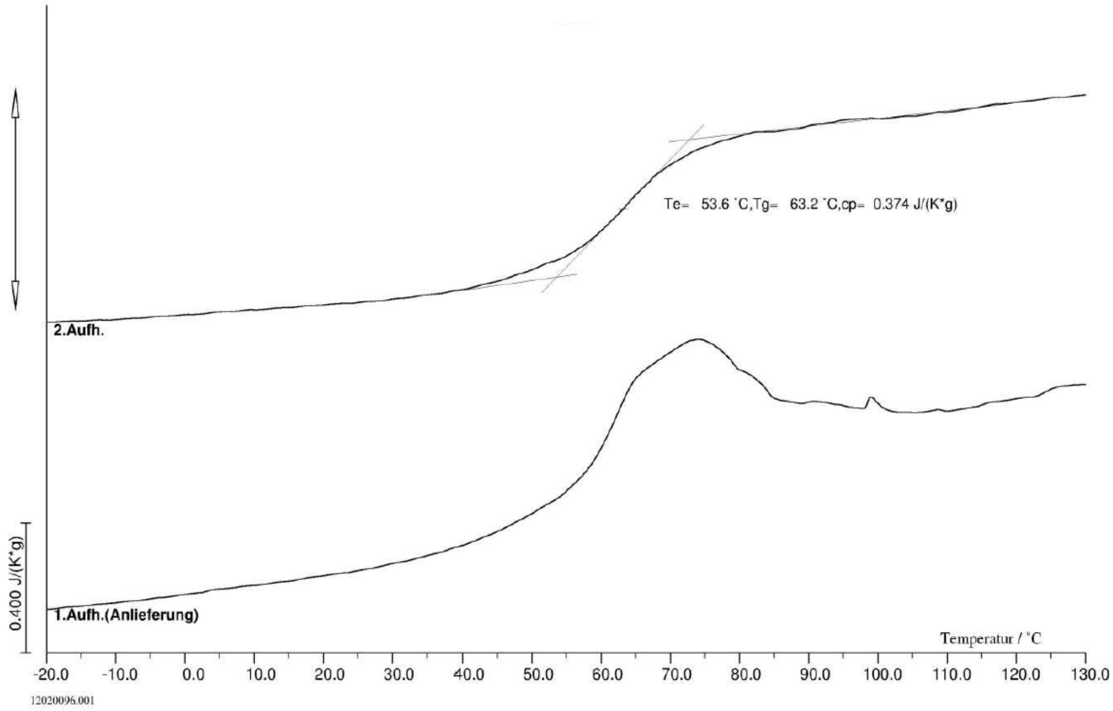

Figure S2: Differential scanning calorimetry (DSC) graph for the determination of polymer glass transition temperature (T_g) of the particles used in the experiments reported. The T_g was measured to be 63.2°C.

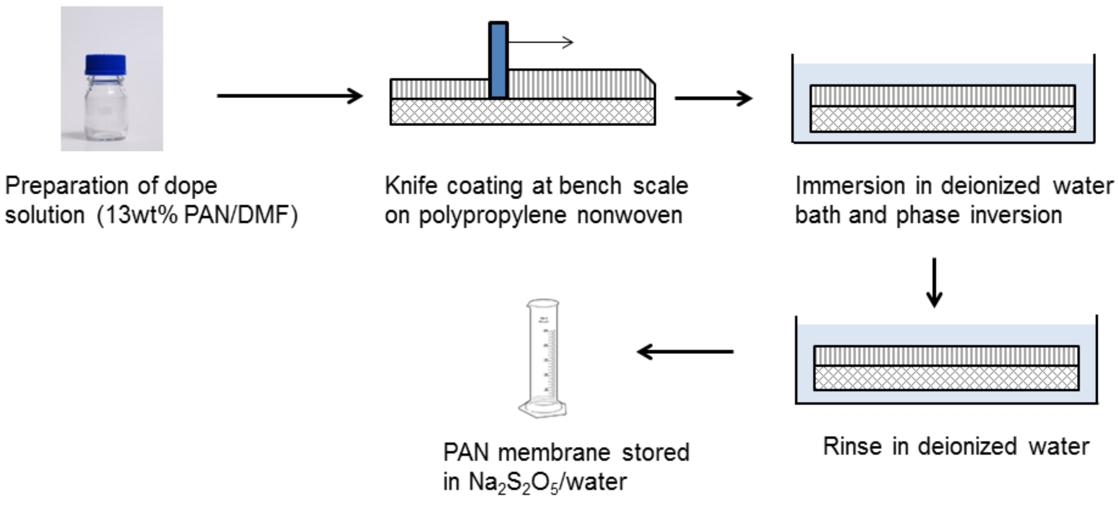

Figure S3: Schematic representation of the PAN preparation process via phase inversion.

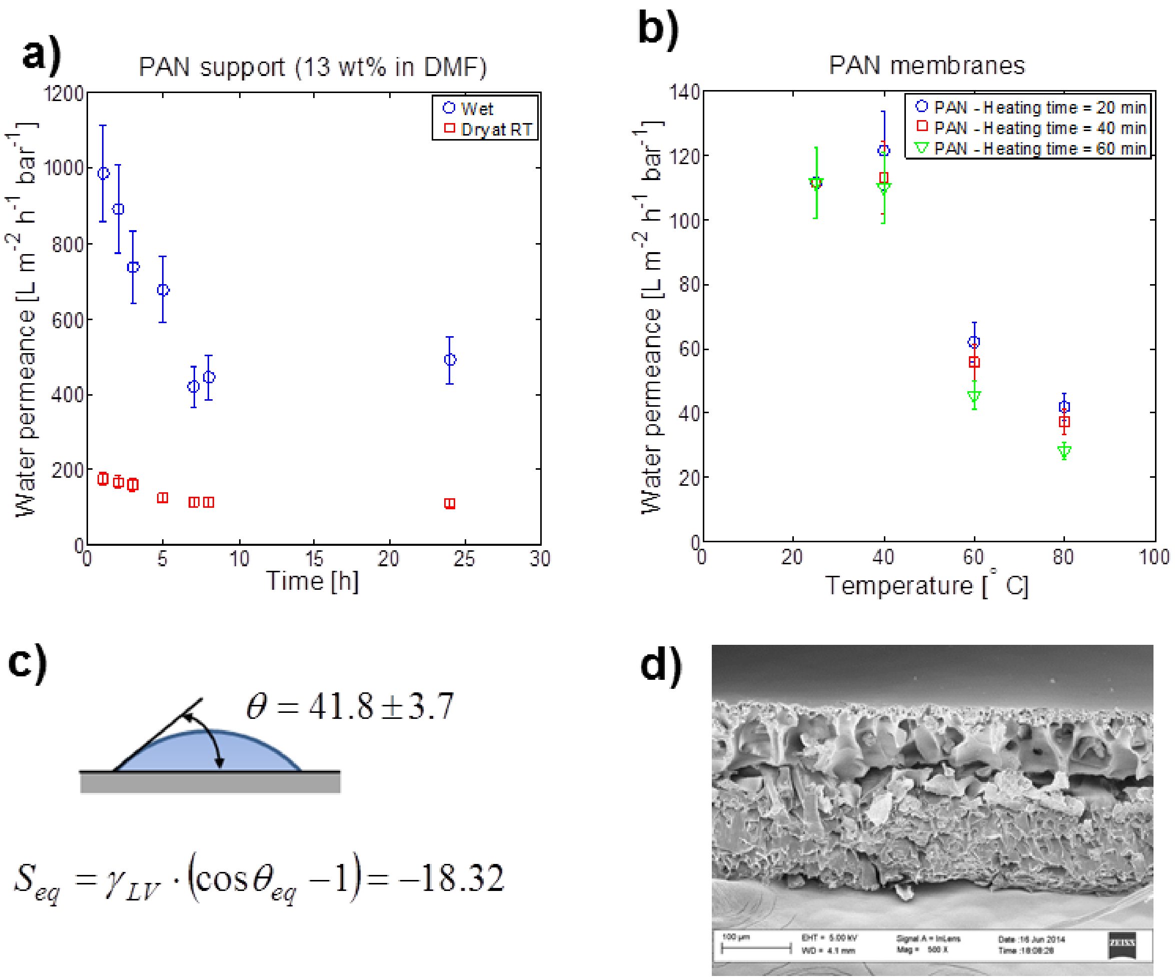

Figure S4: (a) Water permeance of PAN supports in wet and dry conditions (dry = drying at room temperature for at least 24 h). (b) Water permeance of PAN supports dried at room temperature for at least 24 h and then heated in the oven at different temperatures for different times. (c) Contact angle, θ and spreading coefficient, S_{eq}, of PAN support after drying (at room temperature for at least 24 h). The contact angle on the wet PAN support was not measurable ($\theta \to 0$). (d) Cross-section SEM of PAN UF support.

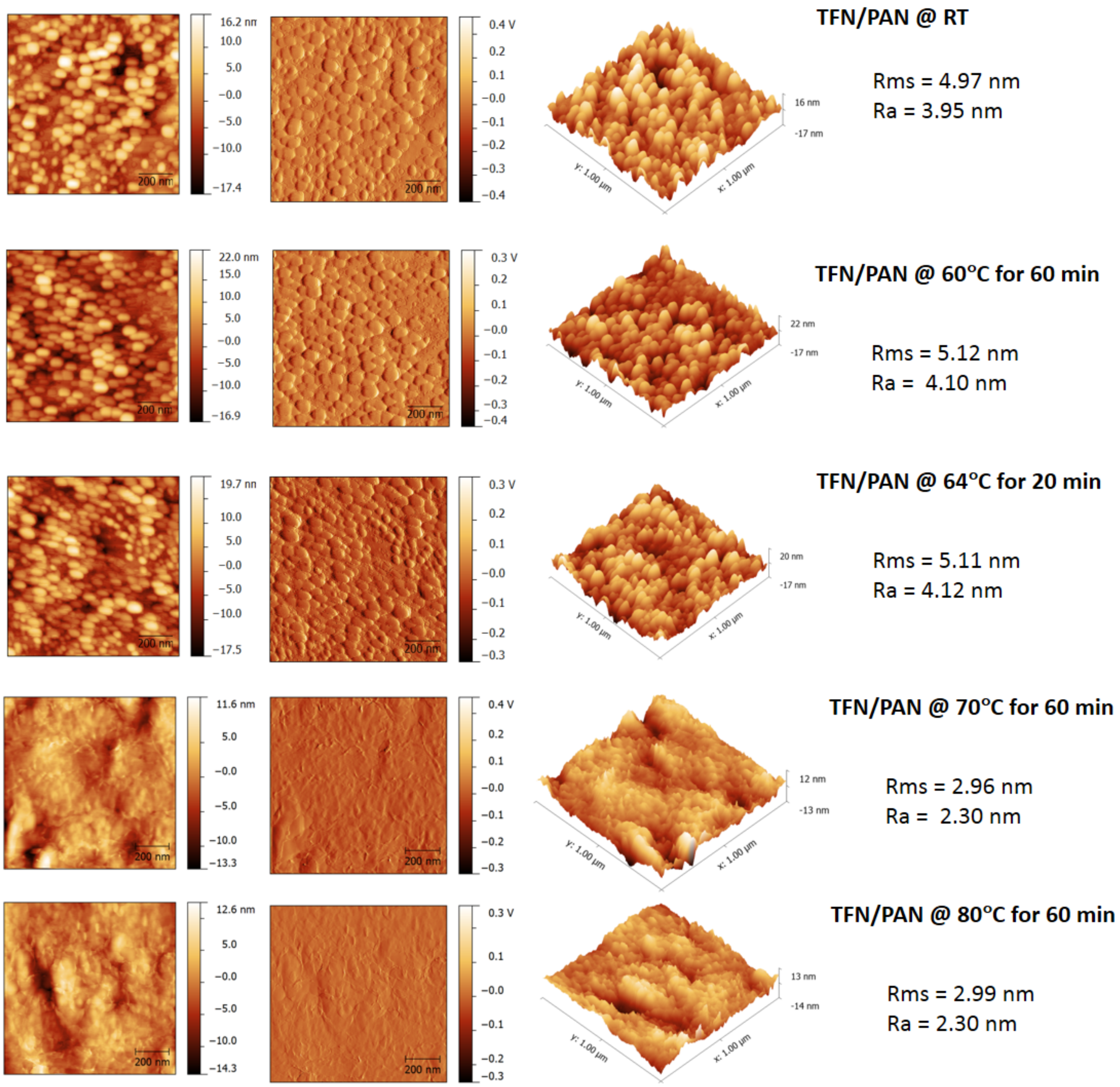

Figure S5: AFM surface images of TFN membranes at different magnifications on porous PAN support. The films were prepared via wire-wound rod coating with a nanoparticle surface loading in water of 66 mg_{NP} m^{-2}. The membranes were dried at room temperature for at least 24 h and then heated in the oven at different temperature for different times.

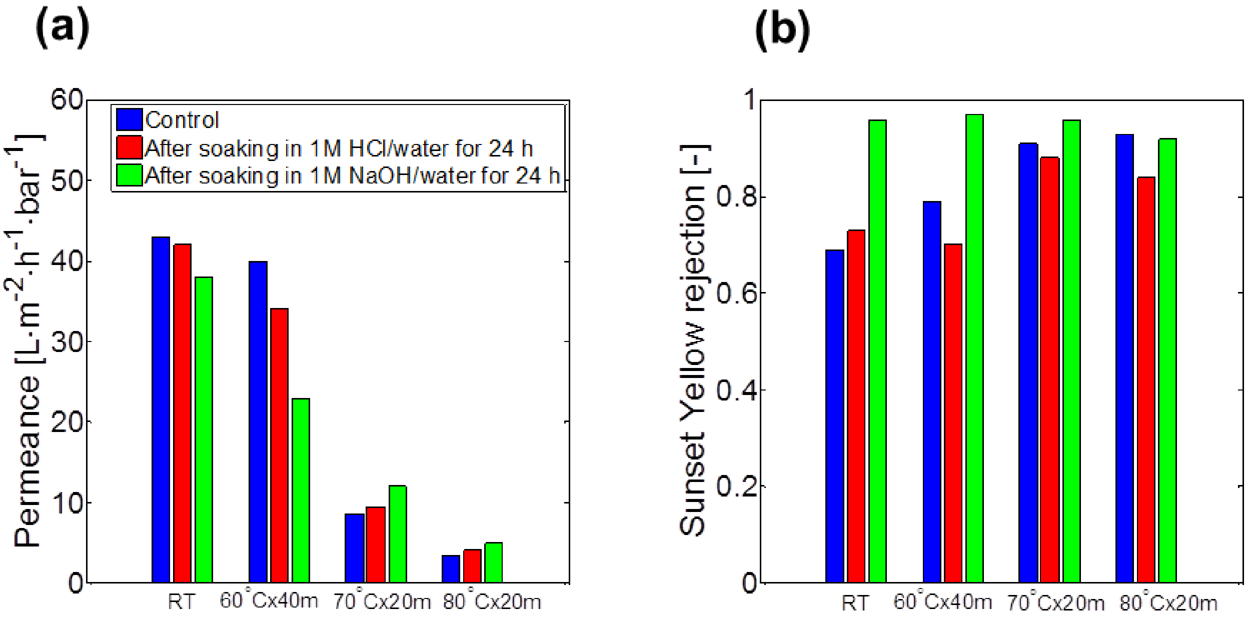

Figure S6: Performance of TFN membranes on porous PAN support with Sunset Yellow dye in water (20 mg L^{-1}). The films were prepared via wire-wound rod coating with a nanoparticle surface loading in water of 66 mg_{NP} m^{-2}. The membranes were dried at room temperature for at least 24 h and then heated in the oven at different temperatures for different times. The control membrane was tested without further treatment, while different discs of the same batch were tested after soaking in 1M HCl/water and 1M NaOH/water, respectively, for 24 h. Prior to testing, the membranes were thoroughly rinsed with water, to restore a neutral pH. (a) Water permeance; (b) Sunset Yellow rejection.

As clear from Figure S6, the membrane had slightly lower permeance than the control membrane when heated below Tg, while it had slightly higher permeance when heated above Tg, after both acid and basic soaking. Rejection of Sunset Yellow dye did not change significantly after soaking in the acid solution, with respect to the control membrane, while the dye rejection improved after soaking in the basic solution. This could be due to an effect of the support hydrolysis in NaOH solutions or to an effect of additional charge taken by the membrane after soaking in the basic environment. A thorough investigation on the role of the support and the possible extra charge upon soaking on the final membrane performance is beyond the scope of this paper

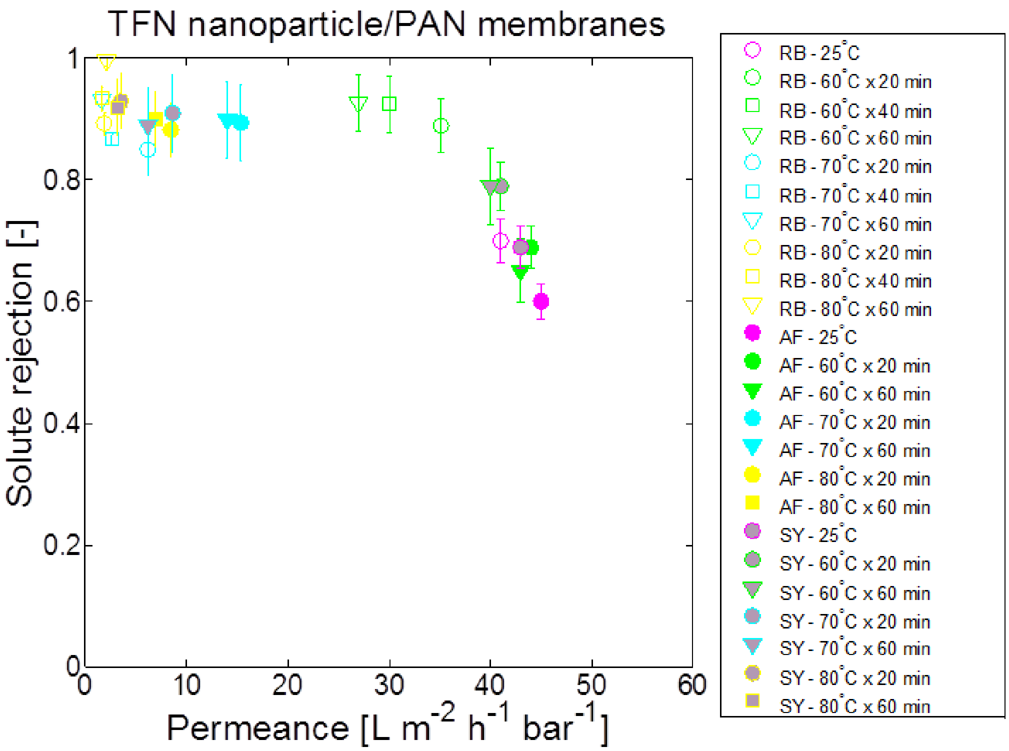

Figure S7: Performance of TFN membranes on porous PAN support with different dyes in water (20 mg L^{-1}). The films were prepared via wire-wound rod coating with a nanoparticle surface loading in water of 66 mg$_{NP}$ m^{-2}. The membranes were dried at room temperature for at least 24 h and then heated in the oven at different temperature for different times.

Table S1: Parameters from modelling membrane performance with dyes: $Y = \frac{(Y_0 - Y_\infty)}{2}\left(1 - tanh\frac{\Delta T}{T - T_g}\right)$. T *is the temperature and* Y *the response (permeance or rejection).*

	$\mathbf{Y}_0$	$\mathbf{Y}_\infty$	Δ**T**
**Rose Bengal/water**			
Water permeance	45.89	6.69	3.63
Permeance t = 20min	42.11	2.15	5.26
Permeance t =40min	40.68	0.00	5.99
Permeance t = 60min	39.54	0.00	6.38
Rejection t = 20min	0.68	0.94	15.46
Rejection t = 40min	0.69	1.00	21.59
Rejection t = 60min	0.72	1.00	12.46
**Acid Fuchsin/water**			
Permeance t = 20min	46.56	11.15	3.99
Permeance t = 60min	46.56	11.15	3.99
Rejection t = 20min	0.62	0.91	5.70
Rejection t = 60min	0.60	0.91	4.24
**Sunset Yellow/water**			
Permeance t = 20min	44.00	5.32	3.73
Permeance t = 60min	43.44	4.02	3.62
Rejection t = 20min	0.69	0.96	10.81
Rejection t = 60min	0.69	0.95	13.92

Table S2: p-value (S = significant; NS = non-significant) of temperature and time on solvent permeance and solute rejection by Design Expert 8 based on a linear model. p-value represents the statistical significance of the parameter (temperature and time) on the response (solvent permeance and solute rejection) and is significant if lower than 0.05. The third column informs if the overall mean is a better descriptor.

	**Temperature**	**Time**	**Is overall mean better?**
**Rose Bengal/water**			
Permeance	0.002 (S)	0.710 (NS)	no
Rejection	0.004 (S)	0.235 (NS)	no
**Acid Fuchsin/water**			
Permeance	0.030 (S)	0.917 (NS)	no
Rejection	0.012	0. 939 (NS)	no
**Sunset Yellow/water**			
Permeance	0.028 (S)	0.927 (NS)	no
Rejection	0.008 (S)	0.773 (NS)	no
**Raffinose/water**			
Permeance	0.029 (S)	0.644 (NS)	no
Rejection	0.025(S)	0.050 (NS)	no
**$MgSO_4$/water**			
Permeance	0.005 (S)	0.219 (NS)	no
Rejection	0.003 (S)	0.724 (NS)	yes

A.1 Nanoparticle core-shell nature

The core-shell structure can be explained by varying reactivity parameters of the monomers used for nanoparticle synthesis. The polymerization of the particles is a radical emulsion polymerization, which starts from an emulsion of butadiene droplets in water, stabilized with a surfactant, together with styrene monomers, trimethylolpropane trimethacrylate TMPTMA (crosslinker), and 2-hydroxyethyl methacrylate HEMA (OH-functionalizer). According to the monomer reactivity parameters, [?] butadiene initially reacts with itself, then with styrene and TMPTMA, and only finally with HEMA. Hence, butadiene and styrene are consumed in the first stages of the reaction, when the core is formed (assuming that the droplet is like a closed reactor with no material exchange). Because HEMA is the slowest reactant, it is more likely to be built into the polymer chain at the

end of the process, and so may be present as protruding chains from the particle core. Since the crosslinker TMPTMA is consumed relatively quickly, the outer regions of the particle are assumed to have a lower crosslinking degree than the core, leading to slightly softer mechanical properties and higher polymer chain mobility.
